# Spatial alignment and the motion bridging effect: Reversals in the direction of an illusory rotation

**DOI:** 10.1177/20416695251318945

**Published:** 2025-03-17

**Authors:** Maximilian Stein, Robert Fendrich, Uwe Mattler

**Affiliations:** 9375 University of Goettingen, Goettingen, Germany

**Keywords:** motion aftereffects, motion perception, unconscious perception, apparent motion, visual illusion

## Abstract

When a stationary ring of points precedes or follows an “inducing ring” of points that spins so rapidly it appears to be a steady outline circle the stationary ring often appears to momentarily rotate in the direction of the inducing ring's spin. In previous studies of this “motion bridging effect” (MBE) the start and stop positions of the inducing ring points were spatially aligned with the points of the stationary ring. Here we report that as these start and stop positions are progressively displaced across the spaces separating the points of the stationary test ring the MBE direction congruency effect decreases and then reverses, so that the illusory rotation is predominantly opposite to the direction of the inducing ring spin. This reverse congruency effect peaks when the points of the inducing ring start and stop midway between the points of the stationary test ring, with congruency returning as further displacements bring the point positions back into alignment. We conclude that the MBE is not only determined by the inducing ring's rotation direction, but also by an interaction between the inducing and test ring points at the moment the inducing ring starts or stops. We consider various ways of accounting this effect. Explanations based on direction cuing by apparent motion steps, the motion aftereffect, and biphasic impulse responses are ruled out. A speculative explanation based on perceptual heuristics that interpret the competing motion direction signals generated by a transformation of contour segments of the spinning ring into the points of the stationary ring (or vice-versa) is proposed.

## How to cite this article

Stein, M., Fendrich, R., & Mattler, U. (2025). Spatial alignment and the motion bridging effect: Reversals in the direction of an illusory rotation. *i–Perception*, *16*(0), 1–25. https://doi.org/10.1177/20416695251318945

The motion bridging effect (MBE, Mattler & Fendrich, 2010) is an illusion in which a rapidly rotating ring of points (*the inducing ring*) is preceded or followed by a stationary ring of points (*the test ring*). The stimulus sequence produces an illusory movement of the test ring points that is mainly perceived to be in the direction of the inducing ring spin. This occurs although the speed of the inducing ring is so high that its direction cannot be distinguished when it is presented on its own: all that is seen in this case is a continuous outline circle (Mattler & Fendrich, 2010; Stein et al., 2019, 2020). This percept of a continuous outline with no consciously visible motion can be attributed to visual persistence: at the high inducing ring velocities that produce the MBE all point positions on the circumference of that ring are refreshed at rates that exceed the human flicker fusion threshold (e.g., 125 Hz with 16 rotating points and a velocity of 2,250°/s).

Two approaches have been proposed to explain the MBE. One is the velocity updating hypothesis of Mattler and Fendrich (2010). They propose that although it is not consciously visible the rapid rotation of the inducing ring is retinally encoded and conveyed to motion sensitive neurons in human visual systems. It is further conjectured that as the activity of these neurons dies down when the stationary test ring suddenly replaces the spinning inducer (or vice-versa) they pass through a neural transition stage where slower motion velocities are represented. These transitional signals are conveyed to the test ring, enabling perceptual continuity by bridging the sequential stimulus states and generating the MBE. Evidence favoring this hypothesis comes from the phenomenological reports of participants, who often describe a percept of deceleration when the test ring follows the inducing ring. When the test ring is presented first, many participants report a corresponding acceleration of the test ring (Mattler & Fendrich, 2010), which can be attributed to a transitional activity phase during the buildup of a neural representation of the high velocity rotation. Additional evidence that the inducing ring provides directional information comes from priming effects. When an inducer with an invisibly rapid rotation is followed by a clearly rotating ring, response times to report the second ring's direction are shorter when the initial and second ring's directions are congruent than when they are incongruent, indicating that the direction of the inducer has been encoded (Mattler & Fendrich, 2007).

An alternative approach to the MBE was proposed by Stein et al. (2019). This approach speculates that the transition from the visually unbroken inducing ring outline to the dotted test ring produces a transformational apparent motion (Tse et al., 1998), perhaps related to the filling in (impletion) process proposed by Downing and Treisman (1997) as an explanation of the line motion illusion. This account predicts that a test ring rotation in some direction should be perceived even if the spinning inducing ring is replaced by a truly continuous stationary outline circle. Mattler et al. (2021) have confirmed this prediction, demonstrating that the test ring rotation is not necessarily dependent on motion signals generated by the inducer. However, the direction congruency effect, which is a defining characteristic of the MBE, demonstrates that the inducer motion does contribute to the test ring motion when it is present. To reconcile these observations, Stein et al. (2019) and Mattler et al. (2021) posit that the MBE reflects the joint action of two processes: an apparent motion process that generates the illusory test ring spin and a direction encoding process that biases the perceived direction of the illusory test ring spin despite the fact that this direction is not detectable in the inducer itself.

Mattler and Fendrich (2010) demonstrated that the MBE is highly dependent on the spatial proximity of the inducing ring and test ring. The MBE congruency effect quickly declined when the outline of the test ring was displaced by small spatial distances from the outline of the inducing ring. When the test and inducing rings were presented spatially superimposed their rotation directions were congruent on better than 75% of the trials. When a test ring with a diameter of 6° of visual angle followed an inducing ring with a diameter 5° the test ring rotation only matched the direction of the inducing ring on 55% of the trials. Similarly, a 1° upward offset of the test ring reduced the MBE congruence effect to 58%. Slightly larger (3°) size and position shifts effectively eliminated the congruence effect. Small spatial separations between the inducing and test rings also degraded the percepts of motion, though to a somewhat lesser extent than the congruency effect: a test ring rotation was reported on close to 100% of the trials when the inducing and tests rings were spatially superimposed, but no-motion percepts were reported only 32% of the time with a 3° test ring expansion.

However, Mattler and Fendrich only considered spatial separations between the entire test and inducing rings. In all previous studies of the MBE the start and stop positions of the inducing ring points have matched the positions of the subsequent test ring points. In the present study, we evaluated the effect of varying the spatial relationship between the inducing and test ring points when the ring circumferences completely overlapped. This was done by shifting the initial and final positions of the inducing ring points relative to the test ring point positions.

### Overview

In the present study, we report two experiments that investigated the dependence of the MBE on the start and stop positions of the inducing ring. In experiment 1, we displaced the start and stop positions of the inducing ring points in multiple steps across the spaces separating the points of the subsequently presented test ring. Experiment 2 was similar to experiment 1, but we reversed the presentation order of the two rings so that the test ring preceded the inducing ring.

### Participants

Participants were students at the University of Göttingen with an average age of 22.6 years. They were paid at €7 per hour or received course credits. All were tested with a Landolt ring chart prior to their first session and found to have normal or corrected to normal vision. Twelve students completed four 1-h sessions in each experiment. No student participated in both experiments.

### Apparatus

Displays were presented on an analog HAMEG HM 400 cathode-ray oscilloscope controlled by a PC with a 12-bit digital-to-analog converter. The 8 × 10 cm oscilloscope screen was customized with a fast P15 phosphor (50 µs luminance decay time to 0.1%). Participants sat in a dark room with their head positions stabilized by a chin and forehead rest 57 cm from the oscilloscope screen.

## Experiment 1

### Stimuli and Procedure

The stimuli were similar to those used in Stein et al. (2019). A rotating ring and a stationary ring were displayed. Both rings were 7.5° of visual angle in diameter and constructed from 16 equally spaced luminous points. These points were slightly blurred because we previously found this strengthened the illusory motion percept being studied. The position of the points could be adjusted in increments of 0.25 angular degrees, allowing them to be placed in 1,440 potential positions along the ring circumference. The inducing ring was rotated by advancing all of its points every millisecond by a specified number of positions so that it was updated with an effective frame rate of 1,000 Hz. Angular clockwise or counterclockwise rotations at velocities of 250, 750, 1,500, and 2,250°/s were respectively produced by sequential point position advances of 1, 3, 6, and 9 steps. At the higher rotation speeds (above 250°/s) the inducing ring appeared to be a continuous solid circle. To eliminate visible switching artifacts, the oscilloscope electron beam was turned off during its transition between the successive point positions. The time-averaged brightness of inducing ring points was 0.017, 0.050, 0.099, and 0.149 cd/m^2^ at the 250, 750, 1,500, and 2,250°/s velocities respectively, and the test ring point brightness was 1.50 cd/m^2^ on a dark background. The methods required to obtain these brightness measurements are described in Stein et al. (2019). Data reported in Stein et al. (2019) indicate the velocity dependent differences in luminance were unlikely to have any impact on the MBE.

**Figure 1.**
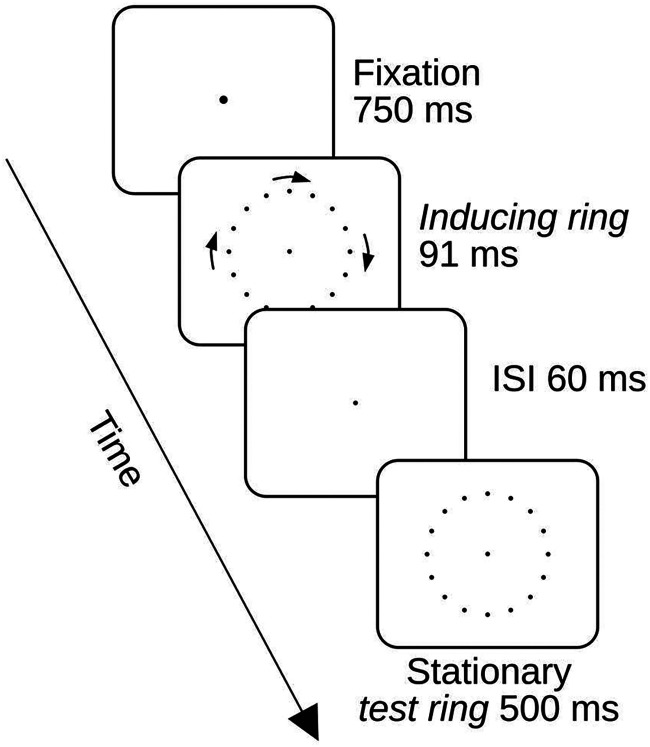
Display sequence in experiment 1. The inducing ring rotated either clockwise (as illustrated in the figure) or counterclockwise. In the standard session, no test ring was presented in half of the trials and the inducing ring start and end point positions coincided with the test ring point positions when the test ring was shown. In the displacement conditions, the test ring was always presented and the start and stop positions of the inducing ring points were shifted across the space between the test ring points. In the actual experimental displays, the points were always bright on a dark background.

The display sequence in both an initial *Standard Alignment* session and three subsequent *Displacement Sessions* is shown in Figure 1. Participants were instructed to maintain their gaze on a central ﬁxation point during the trials. This fixation point brightened for 750 ms at the start of each trial to indicate that the inducing ring was about to appear. The inducing ring was presented for 91 ms. In the conditions in which the test ring was presented it followed the inducing ring after a 60 ms interstimulus interval (ISI) and remained visible for 500 ms.

Participants made a forced choice report of whether a clockwise or counterclockwise ring rotation had been perceived by pressing the arrow keys of a conventional computer keyboard, with the right arrow indicating a clockwise rotation and the left arrow a counterclockwise rotation. They were instructed to guess, following any intuitions they might have, if no rotation or rotation with a clear direction was seen. Because we wanted participants to take the time they needed to consider such intuitions we did not ask them for speeded responses. Reports were recorded starting 300 ms after the offset of the test ring or 300 ms after the offset of the inducing ring when no test ring was presented. No feedback was given about the correctness of responses.

In the *Standard Alignment* condition, the rotation of the inducing ring always started and stopped with points placed at the positions where the test ring points were presented. Fourteen blocks of 48 trials were run in thus session, with the first two treated as practice and excluded from data analysis. Blocks in which only the inducing ring was presented were alternated with blocks in which both the inducing ring and the test ring were presented. The angular velocity and the rotation direction of the inducing ring were varied quasi-randomly within each block. The factorial combination of two test ring states (present, absent) and four angular inducing ring velocities (250, 750, 1,500, and 2,250°/s) produced eight experimental conditions. We collected data on 72 trials in each of these conditions: 36 with a clockwise inducing ring rotation and 36 with counterclockwise rotation.

**Figure 2.**
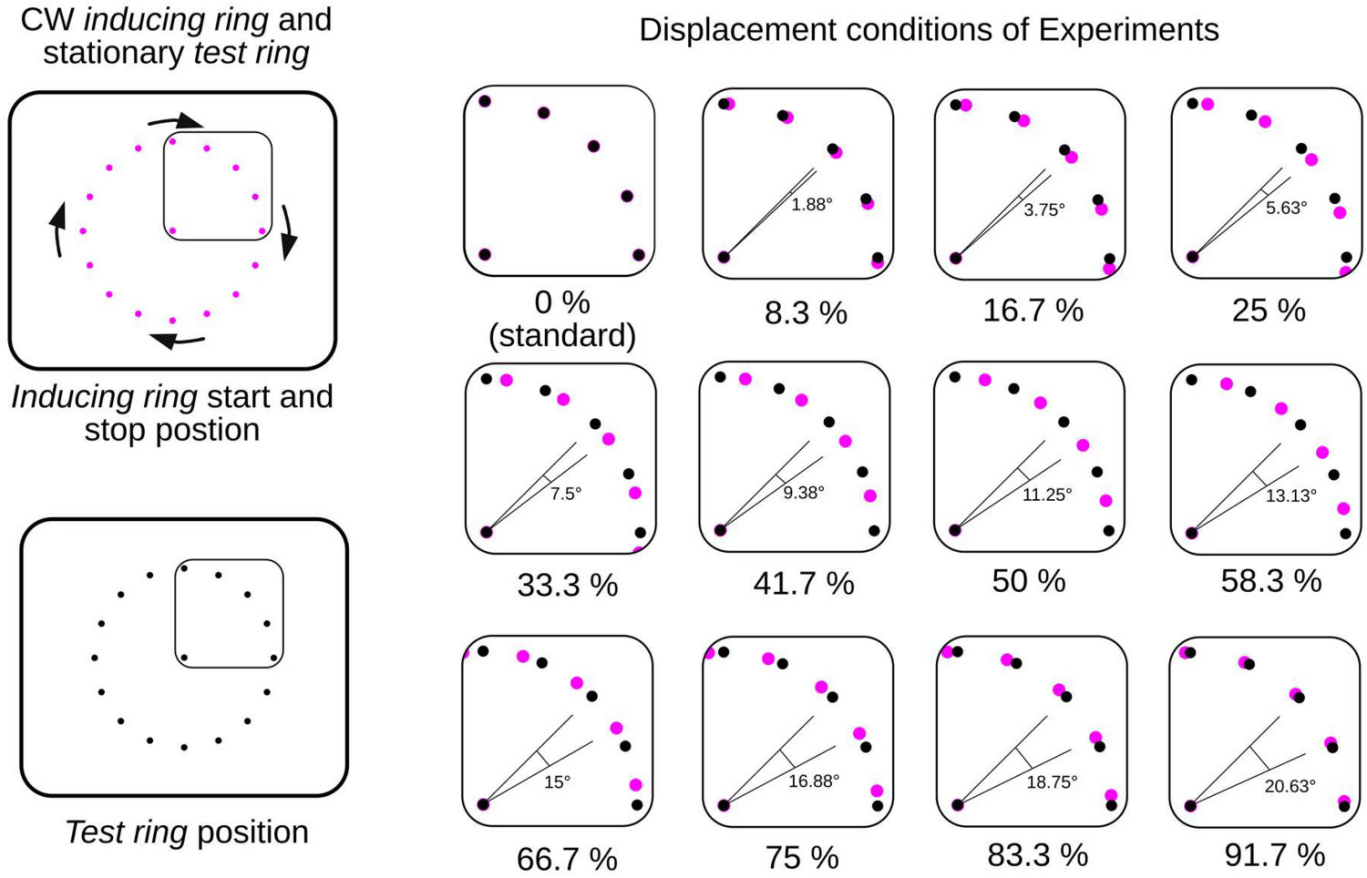
All conditions of the displacement sessions with a clockwise (CW) rotating inducing ring. Test ring point positions are shown in black. The inducing ring start and stop positions were displaced in 11 steps (shown here by the pink dots) across the 22.5° angular distance separating the test ring points. In the actual experimental displays, the points were always bright on a dark background.

In the three subsequent *Displacement Sessions* the lowest velocity (250°/s) was not employed, and the test ring was always presented. The start and stop positions of the inducing ring points was displaced relative to the point positions in the test ring across the 22.5° distance separating the points used to construct these rings in 11 equal steps of approximately 1.88° angular degrees starting with a displacement of 0. These displacements, which are illustrated in Figure 2, were always in the rotation direction of the inducing ring (i.e., clockwise with a clockwise rotation, counterclockwise with a counterclockwise rotation). Nine trial blocks were run in each displacement session, with the first block treated as practice and excluded from data analysis. There were 72 trials in each block. The angular velocity, the degree of displacement, and the rotation direction of the inducing ring were varied quasi-randomly within each block. The combination of 12 displacements (0, 1.88, 3.75, 5.63, 7.5, 9.38, 11.25, 13.13, 15, 16.88, 18.75, and 20.63° across the 22.5° angular point-distance) and three angular inducing ring velocities (750, 1,500, and 2,250°/s) produced 36 experimental conditions. Across sessions there were 48 trials in each of these conditions: 24 with a clockwise inducing ring rotation and 24 with a counterclockwise rotation.

### Statistical Analysis

We used signal detection methods to analyze performance (Macmillan & Creelman, 2005). Hits were defined as “clockwise” responses to a clockwise rotation direction and false alarms as “clockwise” responses to a counterclockwise direction. Hit- and false-alarm rates were estimated separately for each subject in each condition and corrected with the log-linear rule (Hautus, 1995). In the standard alignment sessions, we evaluated *d′* measures of the discrimination ability of participants with repeated measures analyses of variances (ANOVAs). All degrees of freedom were corrected using Greenhouse–Geisser estimates of sphericity but for the sake of readability, the uncorrected degrees of freedom are reported. In addition, Bayes factors for all ANOVAs and *t*-tests were computed using the BayesFactor package (Morey & Rouder, 2024). Note that these Bayes factors were not adjusted for multiple comparisons and were calculated based on non-directional alternative hypotheses.

Note that contrary to classical signal detection analysis the *d′* measures in our analyses do not represent the distance between the means of the noise and signal distributions but the distance between the means of two distributions that correspond to the two motion directions. The value of *d′* is positive when the percentage of “clockwise” responses to a clockwise rotation (hit rate) is higher than the percentage of “clockwise” responses to a counterclockwise rotation (false-alarm rate). Thus, positive *d*′ values indicate that participants perceive a motion direction that is congruent to the rotation of the inducer. Negative values of *d′* indicate a reversal of the relationship between hit- and false-alarm rates. This signifies participants were reporting a predominantly counterclockwise rotation when the inducing ring was in fact rotating clockwise (and vice versa). Negative *d*′ values indicate that participants perceive motion with a direction incongruent to the rotation of the inducer.

Because the effect of the inducing ring displacements on *d*′ was found to have an apparently sinusoidal profile, a sinusoidal function was fitted to each participant's *d′* values at each inducing ring velocity using least squares regression. Variations of *d*′ were measured over the periodic interval that begins and ends with a 0° displacement of the inducing ring start and stop positions relative to the test ring point positions. The mean fit and amplitude of each sinusoidal function was calculated, and amplitude estimates were evaluated across participants with Greenhouse–Geisser-corrected repeated measures ANOVAs.

### Results: The Standard Alignment Session

**Figure 3.**
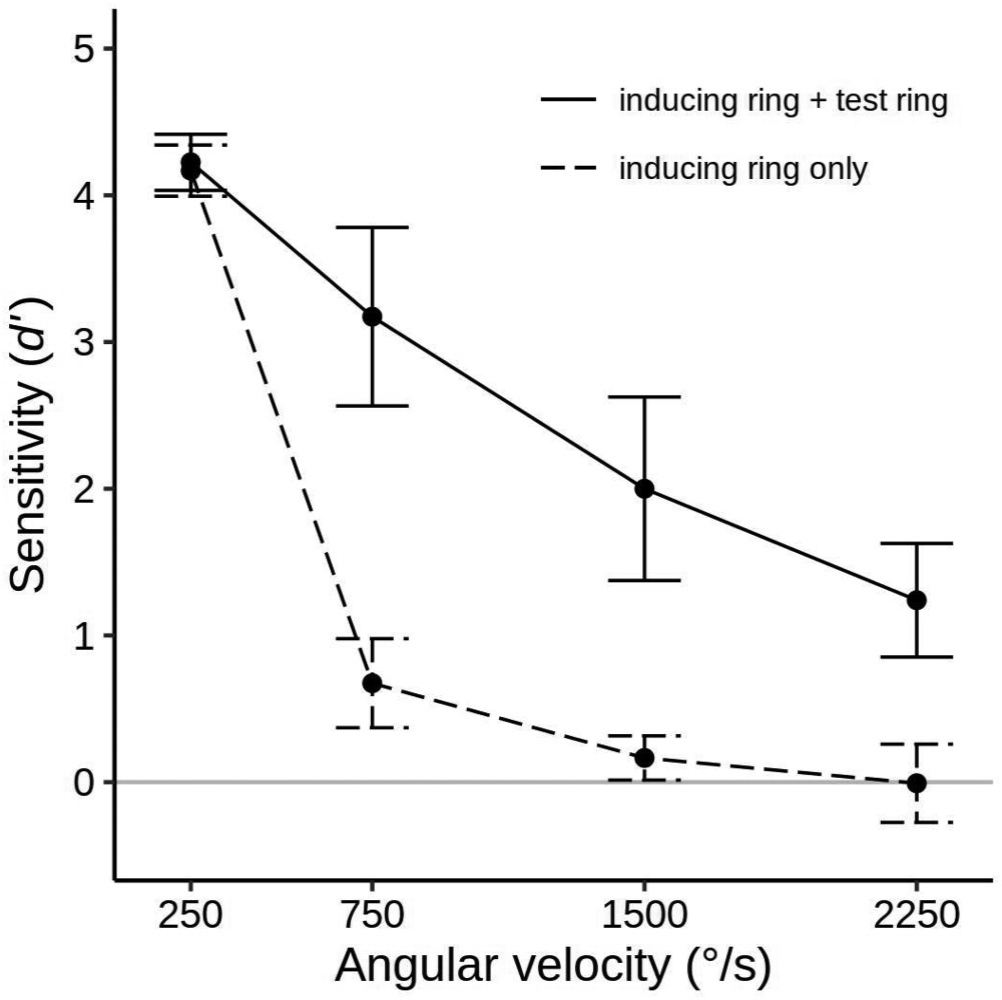
Mean sensitivity (*d′*) for 12 participants in the standard alignment session of experiment 1 for each velocity in the inducing ring only conditions (the dashed line), and the inducing ring + test ring conditions (the solid line). Error bars show 95% confidence intervals. The solid gray line indicates the chance level of accuracy (*d′ *= 0). Data in terms of percent correct performance are given in Table 1.

For the standard alignment session, the mean sensitivity (*d′*) to the inducing ring's direction was calculated for each condition and examined with a two-way 2 × 3 ANOVA that evaluated the effect of presenting the test ring and the inducing ring's angular velocity. We will designate these factors as *Test ring* and *Velocity*. Results and confidence intervals are presented in Figure 3. Additionally, we report mean percent of responses matching the actual inducing ring direction in Table 1.

**Table 1.** Mean percentage of direction reports matching the inducing ring direction in the standard alignment session of experiment 1.

Standard alignment session of experiment 1				
	Angular velocity (°/s)			
Test ring condition	250	750	1,500	2,250
Inducing ring only	99.2	62.6	53.0	50.0
Inducing ring + test ring	99.3	93.2	81.4	72.0

The occurrence of the MBE was confirmed by a higher sensitivity to the inducing ring rotation direction when the test ring was present than when it was absent, *d′ *= 2.66 versus *d′ *= 1.25, *F*(1, 11) = 90.05, *p *< .001, η^2^_G _= .600, *BF_10 _*> 800. There was also a significant main effect of *Velocity*, *F*(3, 33) = 295.69, *p *< .001, η^2^_G _= .851, *BF_10 _*> 800, with mean sensitivity declining as angular velocities increased (*d′ *= 4.20, *d′ *= 1.92, *d′ *= 1.08, and *d′ *= 0.62 for the 250, 750, 1,500, and 2,250°/s velocities, respectively). However, the magnitude and character of this decline was quite different for the two *Test Ring* conditions, producing a significant interaction between *Test ring* and *Velocity*, *F*(3, 33) = 32.81, *p *< .001, η^2^_G _= .379, *BF_10 _*> 800. When the inducing ring was presented alone, performance was high in the 250°/s velocity condition but dropped steeply to near zero or zero at the other three levels of *Velocity* (*d′ *= 4.17, *d′ *= 0.67, *d′ *= 0.16, and *d′ *= −0.01 at 250, 750, 1,500, and 2,250°/s, respectively). In contrast, when the test followed the inducing ring accuracy fell off in a more gradual linear manner with *d′* staying above zero even at the highest level of *Velocity* (*d′ *= 4.22, *d′ *= 3.17, *d′ *= 2.00, and *d′ *= 1.24 at the 250, 750, 1,500, and 2,250°/s velocities, respectively).

The presence of the MBE at each of the inducing ring velocities was evaluated by four paired one-tailed *t* tests that compared the inducer-only and test ring present conditions. At all velocities higher than 250°/s, accuracy rates were significantly higher when the test ring was shown (*t*(11) = 12.62, *p *< .001, Cohen's *d *= 2.64 and *BF_10 _*> 800, for the 750°/s condition, *t*(11) = 6.89, *p *< .001, *d *= 2.22 and *BF_10 _*> 800, for the 1,500°/s condition, and *t*(11) = 5.02, *p *< .001, *d *= 2.41 and *BF_10 _*= 89.1, for the 2,250°/s condition). There was no significant MBE in the 250°/s velocity condition (*p *> .1) because accuracy rates were at ceiling irrespective of the test ring condition.

The improvement in accuracy produced by the test ring presentation can occur even when subjects perform at chance in the inducing ring only condition. To determine if this was the case with the present data, we conducted four one-tailed *t* tests with a Bonferroni-adjusted alpha level of .0125 that compared the performance of participants at each velocity in the inducing ring only condition to a chance level of 50% (*d′ *= 0). Observers easily saw the rotation in the 250°/s velocity condition, and their mean sensitivity was significantly greater than zero at 750°/s, *t*(11) = 4.89 (*p *< .001, *d *= 1.41, *BF_10 _*= 74.7), but at chance at the two highest velocities. When we employed an uncorrected alpha level of .05, performance in the 1,500°/s condition was also slightly better than chance, *t*(11) = 2.41, *p *= .017, *d *= 0.69, *BF_10 _*= 2.19, but not in the 2,250°/s condition, *t*(11) = 0.06, *p *> .525, *d *= 0.02, *BF_10 _*= 0.29.

### Results: Displacement Sessions

**Figure 4.**
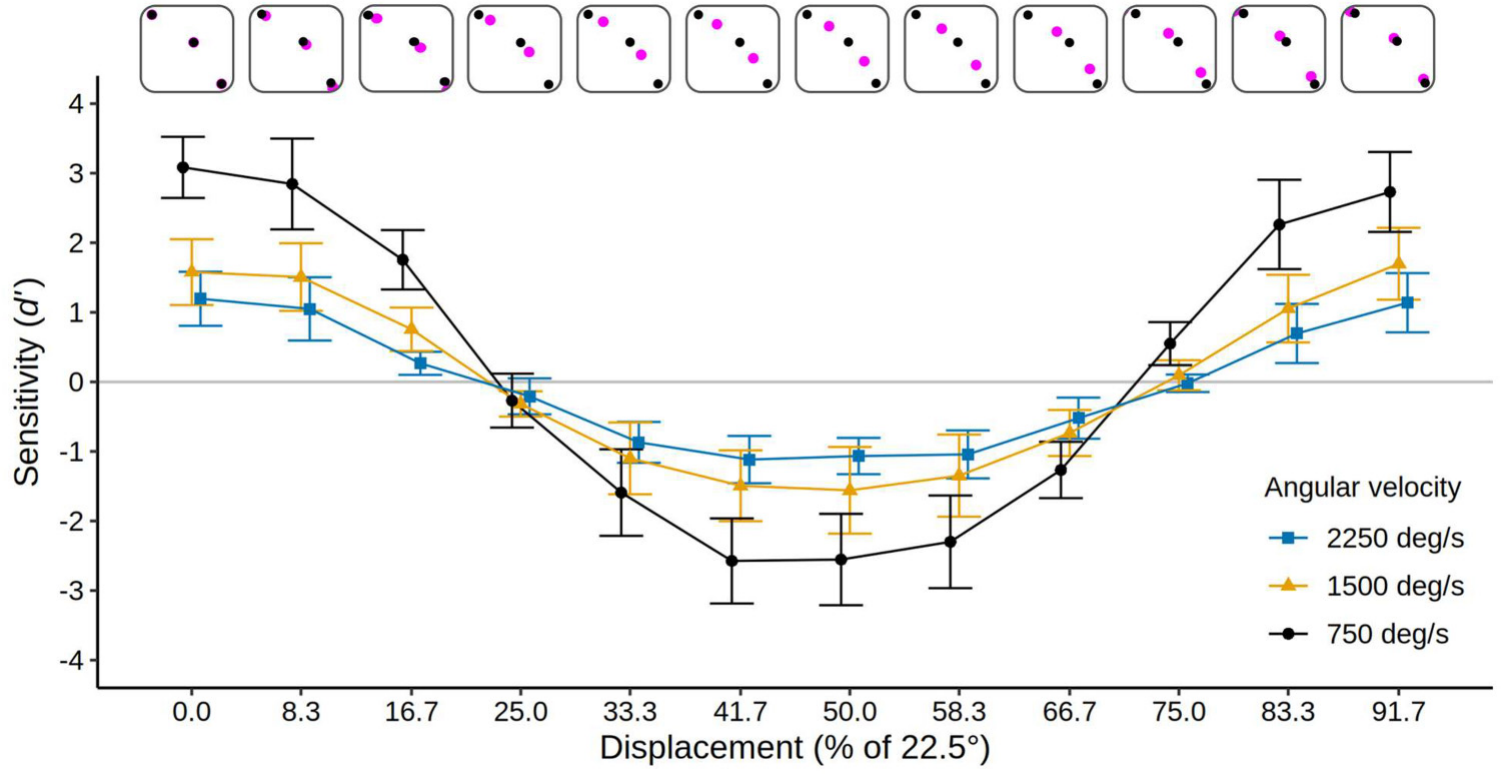
Mean sensitivity (*d′*) for 12 participants in the displacement sessions of experiment 1 for each level of velocity and displacement. The top row illustrates the displacement at each reported level, with black dots indicating the test-ring points and magenta dots indicating the displaced start and end positions of the inducer points. Error bars show 95% confidence intervals. Points and confidence intervals are slightly offset horizontally to improve their visibility. The solid gray line indicates the chance level (*d′ *= 0). Note that positive *d′* values indicate a bias to report motion direction congruent with the actual inducing ring direction, negative *d′* values indicate a bias to report motion opposite to the actual inducing ring direction. Data in terms of percent correct are given in Table 2.

Mean *d′* values and confidence intervals for all the conditions of the displacement sessions are presented in Figure 4. Additionally, Table 2 presents the results as the mean percent of correct reports of the inducing ring direction in each condition. It is evident in Figure 4 that the displacement manipulation had an enormous impact on the MBE. Mean sensitivity is high (*d′ *= 1.95) with a 0% displacement of the inducing ring start/stop positions relative to the test ring point positions (the standard alignment), gradually drops to near zero with a 25% displacement (*d′ *= −0.26), builds in an *inverse* direction (with the reported motion primarily *opposite* the direction of the inducing ring rotation) to a maximum when the displacement is 41.7 to 50% (*d′ *= −1.73 for both), then returns to a normal MBE as further displacements bring the start and stops positions back into alignment with the test ring points (crossing a null effect point again at a displacement of 75%). Thus, the MBE effectively reverses its direction when the inducing ring start and stop points are close to midway or midway between the test ring point positions.

**Table 2.** Mean percentage of reports in inducing ring direction for the displacement sessions of experiment 1.

Displacement sessions of experiment 1				
		Angular velocity (°/s)		
Displacement (as % of 22.5°)		750	1,500	2,250
0		93.8	77.6	71.5
8.3		89.8	75.9	68.8
16.7		79.9	64.6	55.4
25		44.8	44.1	45.7
33.3		23.1	30.2	33.5
41.7		11.1	24.3	30.0
50		11.6	24.1	29.9
58.3		14.4	26.7	30.9
66.7		26.9	35.9	40.1
75		60.6	52.1	49.5
83.3		84.7	68.8	63.0
91.7		90.6	78.8	70.8

*Note*. 50% indicates that the reports of motion direction were unrelated to the inducing ring's motion direction. Values above 50% indicate a bias to report motion direction congruent with the inducer's rotation direction, and values less than 50% indicate a bias to report motion direction opposite to the actual inducing ring direction.

 As noted above, we modelled the data with a sinusoidal function that reproduces the change of *d′* over the various levels of displacement. The high mean fit between the computed sinusoids and participant's *d′* measure of discrimination ability (*R*^2^* *= .96, .88, and .85 at velocities of 750, 1,500, and 2,250°/s, respectively) supported the suitability of this procedure. The amplitude of the fitted sinusoids indicates the strength of the positive and negative MBEs. The amplitude of each subject's sinusoid in each velocity condition was therefore examined with a one-way ANOVA with *Velocity* as the independent variable. The effect of *Velocity* was highly significant, *F*(2, 22) = 111.26, *p *< .001, η^2^_G _= .486, *BF_10 _*> 800, with the sinusoid amplitude declining as velocity increased (mean amplitudes were 3.04, 1.72, and 1.22 at velocities of 750, 1,500, and 2,250°/s, respectively), confirming that velocity modulates the strength of the MBE. As can be seen in Figure 4, an increase in inducer velocity led to a reduction in both, the forward and reverse biasing of the test ring motions, bringing performance closer to chance in both cases.

### Summary and Discussion

In the standard session of experiment 1, we replicate the MBE and its dependence on angular velocity (Mattler & Fendrich, 2010; Stein et al., 2019). When the rapidly rotating inducing ring was presented alone, at high velocities participants perceived a stationary outline and their ability to discriminate rotation direction dropped to the chance level. When the test ring followed the inducing ring, performance was substantially above chance at all velocities because participants perceived an illusory rotation of the test ring in the same direction as the inducing ring had rotated.

Previous investigations have proposed that this occurs because the rotation direction of the inducing ring, although not consciously visible, is registered by the visual system and subsequently affects the direction of the illusory motion. However, the displacement sessions of experiment 1 demonstrate that the perceived direction of motion is highly dependent on the inducing ring start and stop positions. The MBE decreases when these positions are displaced from the test ring point positions, inverts when the displacement is greater than 25% of the inter-point distance, and reaches a maximum negative value when the start and stop points are midway between the test ring points. This reversal is difficult to reconcile with an account of the MBE that posits the direction of the inducing ring rotation is simply encoded and transferred to the test ring. If the direction of the MBE is solely determined by stored information regarding the inducing ring's rotation direction one would not expect the start and stop positions of the inducing ring to have any influence on the direction of the illusory motion. The fact that these positions profoundly effect on the direction of the illusory motion argues this direction is determined not by stored information but the interaction between the inducing and test ring points when the transition between these rings occurs. In our final discussion we address some alternative ways that interaction might occur.

The weakening of both the forward and reverse MBE congruency effects as the inducing ring velocity increases can be attributed to a progressive loss of directional rotation information. In the experiments reported here (as in previous MBE investigations) congruency effects demonstrate that this directional information is derived from stimuli that rotate at velocities so high they would not be expected to be registered as moving by the human visual system, and in fact are not consciously perceived as moving. This is the case because processing these rotations entails the detection of periodic luminance modulations that occur at rates (66.6 Hz with the 1,500°/s rotation, 100 Hz with the 2,250°/s rotation) that exceed most estimates of the human critical flicker fusion frequency. While subject to substantial observer and display dependent variations, this rate has generally been found to be less than 60 Hz (Kelly, 1972). In a study that specifically addresses the effect of temporal frequencies on the ability of observers to judge the direction of drifting sinusoidal gratings, Burr and Ross (1982) found an even more pronounced dependency: discrimination abilities began to deteriorate at temporal frequencies higher than 10 Hz, and at frequencies higher than 30 Hz no motion percepts were seen. See Stein et al. (2019) for a more extensive presentation of these limitations. A decline in the registration of motion direction information as the inducer velocity increases is therefore unsurprising: what is remarkable is that this information is registered at all at the highest velocities we employed. It should be noted that the relevant processing limitations involve not only the encoding and storage of motion signals per se but also the derivation of directional information by comparisons of the strength of the persisting retinal activity at different locations on the inducing rings circumference, since the more rapid the inducing ring rotation the smaller these persistence strength differences will be, and with a 1,500°/s velocity they are already too small to be consciously discernible.

Since the direction of the MBE's congruency effect depends on the alignment of the inducing ring and test ring points, and these rings must be compared across the ISI in our investigations, the effect of this ISI's duration becomes a matter of interest. The ISI was not manipulated in the current experiment. It was, however, investigated in two previous studies (Mattler & Fendrich, 2010; Mattler et al., 2021) where an inverted U-shaped function was found, with a positive congruency effect increasing as the ISI was increased from 0 to 60 ms, then a relatively steady congruency effect until the ISI reached 90 ms, then a decline in the congruency effect, although it was still present with an ISI of 180 ms. The ISI employed in the present experiment were chosen to fall within the maximum congruency interval found in these previous studies. The factors responsible for the inverted U-shaped function remain to be determined, but it is noteworthy that a negative congruency effect has not been found at any ISI evaluated so far.

## Experiment 2

In the classic MBE paradigm, an inducing ring rotation too rapid to be detected when only that ring is shown generates an illusion of motion in the same direction in a subsequent stationary test ring. Observers typically have the phenomenal impression that the test ring is a continuation of the inducing ring in which a rapid spin is rendered visible as it comes to a halt.

In their 2010 paper, Mattler and Fendrich reported a variant of the MBE display in which the test ring preceded the inducing ring. They found this display produced a percept of the test ring launching into motion and accelerating to form a fused solid outline. This variant of the MBE produced an even stronger congruence effect than the classic MBE paradigm. In accord with previous and subsequent observations, when the inducing ring rotation was 1,500°/s and it was presented alone it appeared to be a stationary outline circle and observers performed at chance (51% correct) when asked to report its spin direction, but when it was preceded by the static test ring the illusory test ring rotation was in the inducer direction 92% of the time. To assess the generality of the surprising effect of the displacement manipulations we observed in experiment 1, experiment 2 was performed to determine if these same effects occur when the test ring precedes the inducing ring.

### Method

**Figure 5.**
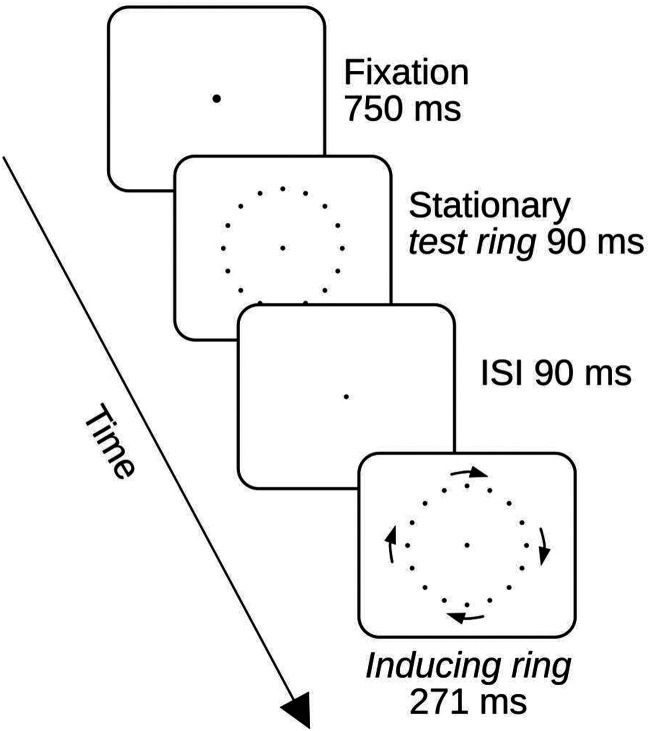
Display sequence in experiment 2. The inducing ring rotated either clockwise (as shown in the figure) or counterclockwise. In the standard alignment session, no test ring was presented in half of the trials. When it was presented, the inducing ring start and end point positions coincided with the test ring point positions. In the displacement sessions the test ring was always presented and the start and stop positions of the inducing ring points were shifted across the space between the test ring points. Note, in the actual experimental displays, the points were always bright on a dark background.

The stimuli, design, and methods of experiment 2 were modeled closely after those of experiment 1 with the order of the test and inducing rings reversed, as illustrated in Figure 5. The test ring duration, ISI, and inducer duration were adjusted to values previously found to produce reliable direction congruency when the test ring preceded the inducing ring (Mattler & Fendrich, 2010). As in experiment 1, we ran a *standard alignment* session in which the start and stop positions of the inducing ring points corresponded to the position of the test ring points, and three *displacement sessions* in which the inducer start and stop positions were shifted in 11 steps across the 22.5° angular inter-point distance. As in experiment 1, 250, 750, 1,500, and 2,250°/s velocities were presented in the standard alignment condition and 750, 1,500, and 2,250°/s in the displacement conditions, with the test ring presented on half the standard alignment trials and all the displacement trials. Refer to the “Methods” section of experiment 1 for a full description of the procedures.

### Results: The Standard Alignment Session

**Figure 6.**
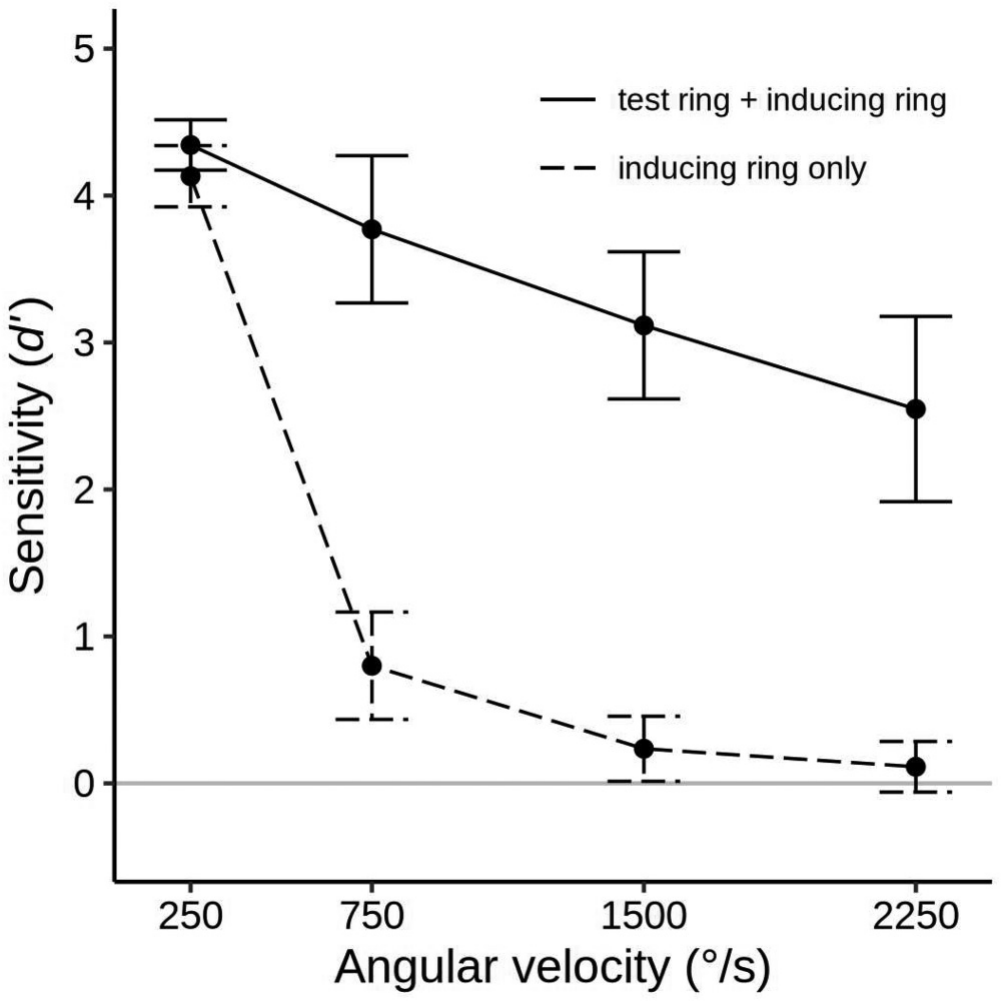
Mean sensitivity (*d′*) for 12 participants in the standard alignment session of experiment 2 for each velocity in the inducing ring only conditions (the dashed line), and the test ring + inducing ring conditions (the solid line). Error bars show 95% confidence intervals. The solid gray line indicates the chance level of accuracy (*d′ *= 0). Data in terms of percent correct are given in Table 3.

Results and confidence intervals for all conditions of the standard alignment session of experiment 2 are presented in Figure 6. Mean percentage of responses in the actual inducing ring direction are presented in Table 3. When the test ring preceded the inducing ring, its effect was similar to that observed when the inducing ring was presented first: mean sensitivity to the inducing ring rotation direction was far higher (*d′ *= 3.44) than when the inducing ring was presented alone (*d′ *= 1.32). A 2 × 3 repeated measures ANOVA with *Test ring* and *Velocity* as the independent variables confirmed the main effect of *Test ring*, *F*(1, 11) = 183.97, η^2^_G _= .770, the main effect of *Velocity*, *F*(3, 33) = 243.48, η^2^_G _= .790, and the *Velocity* by *Test ring* interaction, *F*(3, 33) = 43.73, η^2^_G _= .484 (all *p *< .001, all *BF_10 _*> 800). The interaction reflects the very steep initial decline in performance as velocity is increased in the inducing ring only condition (*d′ *= 4.13, *d′ *= 0.80, *d′ *= 0.24, and *d′ *= 0.11 at 250, 750, 1,500, and 2,250°/s, respectively) versus the inducing ring + test ring condition (*d′ *= 4.34, *d′ *= 3.77, *d′ *= 3.12, and *d′ *= 2.55 at 250, 750, 1,500, and 2,250°/s, respectively).

**Table 3.** Mean percentage of reports in inducing ring direction for the standard alignment session of experiment 2.

Standard alignment session of experiment 2				
	Angular velocity (°/s)			
Test ring condition	250	750	1,500	2,250
Inducing ring only	99.0	64.6	54.6	52.2
Test ring + inducing ring	99.8	97.1	93.2	87.8

In the inducing ring only condition, Bonferroni-corrected one-tailed *t* tests (α* *= .0125) indicated sensitivity was significantly greater than zero in the 250 and 750°/s velocity conditions, with respective *t*(11) values of 43.67, and 4.82 (both *p *< .001, both *BF_10 _*> 10, *d *= 12.61, *BF_10 _*> 800 for the 250°/s condition and *d *= 1.39, *BF_10 _*= 68.18 for the 750°/s condition). Performance did not differ from chance at the 1,500°/s and 2,250°/s velocity conditions, but did exceed 0 in the 1,500°/s condition when an uncorrected alpha was employed, *t*(11) = 2.34, *p *= .020, *d *= 0.68, *BF_10 _*= 2.01 (*t*(11) = 1.44, *p *> .089, *d *= 0.41, *BF_10 _*= 0.66 for the 2,250°/s condition). A comparison of the test ring present and absent conditions using four paired Bonferroni-corrected one-tailed *t* tests (α* *= .125) confirmed the MBE was present at all velocities except for 250°/s, with *t*(11) values of 13.60, 12.68, and 7.64 (all *p *< .001, all *BF_10 _*> 800) and Cohen's *d* values of 4.23, 4.56, and 3.55 for the 750, 1,500, and 2,250°/s velocities, respectively. When employing an uncorrected alpha, there was also a small MBE in the 250°/s velocity condition, *t*(11) = 1.90, *p *= .042, *d *= 0.71, *BF_10 _*= 1.12, although accuracy rates were very high even when the test ring was absent.

### Results: Displacement Sessions

**Figure 7.**
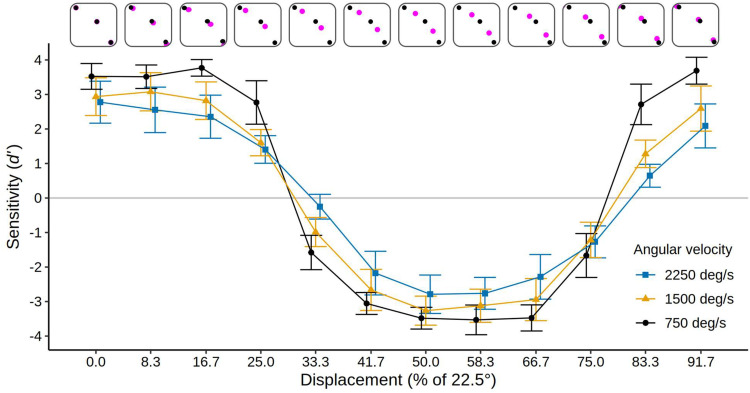
Mean sensitivity (*d′*) for 12 participants in the displacement sessions of experiment 2 for each level of velocity and displacement. The top row illustrates the displacement at each reported level, with black dots indicating the test-ring points and magenta dots indicating the displaced start and end positions of the inducer points. Error bars show 95% confidence intervals. Points and confidence intervals are slightly offset horizontally to improve their visibility. The solid gray line indicates the chance level of accuracy (*d′ *= 0). Note that negative *d′* values indicate a bias to report motion opposite the actual inducing ring direction. Data in terms of percent correct are given in Table 4.

When the test ring preceded the inducing ring, we found a pattern of data similar to that observed in the previous experiment. This is evident in Figure 7 and mean percent correct values are provided in Table 4. Mean *d′* shifts from a positive maximum in the standard alignment condition (*d′ *= 3.08) to a negative maximum at 50% displacement (*d′ *= −3.18) and then back, crossing zero between a displacement of 25% (*d′ *= 1.93) and 33.3% (*d′ *= −0.94) and subsequently between 75% (*d′ *= −1.38) and 83.3% (*d′ *= 1.55). As observed in experiment 1, increases in velocity reduced the magnitude of both the positive and negative values of *d*′ (e.g., *d′ *= 3.52, *d′ *= 2.94, *d′ *= 2.78 at 750, 1,500, and 2,250°/s respectively at 0% displacement and *d′ *= −3.48, *d′ *= −3.26, *d′ *= −2.79 at 750, 1,500, and 2,250°/s respectively at 50% displacement), bringing performance closer to a bias-free level in both cases.

**Table 4.** Mean percentage of reports in inducing ring direction for the displacement sessions of experiment 2.

Displacement sessions of experiment 2				
		Angular velocity (°/s)		
Displacement (as % of 22.5°)		750	1,500	2,250
0		97.2	92.7	89.9
8.3		96.7	92.7	87.7
16.7		98.4	91.3	85.2
25		89.6	77.4	73.8
33.3		23.8	33.2	45.0
41.7		5.7	11.6	16.7
50		3.0	5.4	9.9
58.3		3.3	5.9	9.7
66.7		3.3	8.0	15.3
75		22.6	29.0	28.0
83.3		90.1	72.4	61.3
91.7		97.6	88.5	82.6

*Note*. 50% indicates that reports of motion direction are unrelated to the inducing ring's motion direction. Values above 50% indicate a bias to report a motion direction congruent to the inducer's rotation direction, and values less than 50% indicate a bias to report a motion direction opposite the inducing ring direction.

When we fitted a sinusoid to the change of *d′* over displacement for each subject in each velocity condition and calculated the sinusoid amplitudes, the fits between a sinusoid and the mean *d′* functions were once again excellent (*R*^2^* *= .91, *R*^2^* *= .96, *R*^2^* *= .96 at 750, 1,500, and 2,250°/s, respectively). A one-way ANOVA indicated that the amplitude of the sinusoids varied significantly as a function of *Velocity*, *F*(2, 22) = 30.45, *p *< .001, η^2^_G _= .302, *BF_10 _*> 800, with the amplitude declining as velocity increased (*M* = 4.29, *M* = 3.52, and *M* = 2.98 at 750, 1,500, and 2,250°/s, respectively), confirming both the positive and negative MBE values were modulated by angular velocity.

### Summary

As in previous experiments, when the rotating inducing ring velocity was high and it was presented alone it appeared to be stationary outline circle and participant's ability to discriminate the spin direction was at or near chance. When its velocity was high and it was preceded by the stationary test ring, participants perceived the inducing ring as launching into motion in the same direction as the actual inducing ring rotation before its points fused to form an outline circle. This allowed the inducing ring direction to be reported at substantially above chance levels. As in experiment 1, the MBE congruency effect diminished and then reversed direction as the start and stop positions of the inducing ring points were displaced to positions between the test ring points. The pattern of this reversal is very similar to the pattern observed in experiment 1, with the MBE becoming zero with a 25% displacement, reaching its maximum reverse value at a 50% displacement and then reverting to normal, crossing zero at a 75% displacement. As indicated in the discussion of experiment 1, the weakening of the congruency effects as the inducer velocity increases is attributable to a diminished ability of the visual system to register and process the inducing ring's rotation.

## General Discussion

The MBE has two core attributes: it generates an illusory rotation in a stationary ring of points and biases the direction of that rotation to match that of a previously (or subsequently) shown ring of points that rotates so rapidly its spin direction cannot be discriminated when it is presented alone. In their initial description of the MBE, Mattler and Fendrich (2010) propose it occurs because the rapid rotation of the inducing ring, although not consciously discriminable, is encoded and represented at some visual processing stage. When the transition to the actually stationary test ring occurs, they propose this representation is conveyed to the test ring creating the illusory motion. Subsequent investigations, however, led Stein et al. (2019) to argue that the process that generates the MBE motion percept and the process that acts to bias the direction of that motion's direction are distinct and separable. This dual-process account implies an illusory test ring rotation might be generated even when there is no inducing ring rotation. Mattler et al. (2021) have, in fact, confirmed that this occurs in an illusion they term the *ring rotation illusion* (RRI)—a rotation is seen when a ring of stationary dots replaces a fully drawn static outline circle. Because in the case of the RRI there is no preceding rotation to bias the direction of the illusory rotation it would be reasonable to expect the direction of the illusory motion to be assigned randomly, producing a similar incidence of clockwise and counterclockwise spin directions. Mattler et al. (2021) found, however, the reported direction was, in fact, predominantly clockwise. They suggest this might be due to a greater familiarity of clockwise rotations in the past experience of observers. This suggestion accords with Downing and Treisman's (1997) suggestion that impletion “reflects an implicit…inference which interprets ambiguous stimuli in terms of the most likely real-world state of affairs.” It also accords with a proposal by Hsieh et al. (2005) that transformational apparent motions are, in general, mediated by perceptual heuristics. Adopting this proposal, Mattler et al. (2021) hypothesize that in both the RRI and MBE the motion percept is mediated by a visual heuristic that interprets the sudden replacement of an outline circle by a stationary ring of points as a consequence of a rapidly rotating ring of points coming to a halt. The prior observation by Mattler and Fendrich (2010) that observers often see the test ring rotation as decelerating to a stop is commensurate with this hypothesis. In the case of the MBE, the registered information regarding the inducer rotation would act as a cue that biases the direction of the rotation produced by the visual heuristic.

The results of the present investigation cannot be easily reconciled with the previous accounts of the MBE biasing process: if the direction of the test ring rotation acts to bias the perceived direction of illusory rotation it is difficult to see how this direction could be reversed by the spatial relationship between the test ring point positions and the final inducing ring point positions. While incongruent rotation percepts have occurred before in MBE experiments, they have always been less frequent than congruent percepts and have heretofore simply been regarded as a consequence of the imperfect nature of the biasing process. The viability of this perspective is challenged by the current finding that the incongruent percepts can become *dominant* when the final inducing ring point positions are displaced relative to the test ring point positions.

The present results raise two closely related questions: (1) What is the nature of the signal that generates the reverse motion percepts and (2) why does this signal become ascendant when the test and inducing ring points are misaligned. Below we consider four alternative ways of accounting for the reverse motion percept. These are: (1) an explanation based on apparent motion steps between the inducing and test ring point positions, (2) an explanation based on the classic motion aftereffect (MAE), (3) an explanation based on bivalent motion impulse responses, and (4) an explanation based on the interpretation of competing motion signals by perceptual heuristics. We note in advance that while we feel we can confidently rule out the first three of these approaches, we can only speculate about the viability of the fourth.

### Direction Biasing by Apparent Motion Steps

In all our previous studies of the MBE the inducing ring points have halted (or started) when they were aligned with the test ring points. This was done to eliminate the possibility that apparent motion jumps between the inducing ring and test ring positions would confound the illusory motion percepts. It was deemed possible that such jumps could serve to cue the direction of the illusory rotation even if they were not visually evident.

The pattern of directional dependencies that would be expected if this was occurring bears no resemblance, however, to the pattern actually observed in the displacement outcomes. Consider, for example, the case where the test ring follows the inducing ring and the inducing ring motion is clockwise. Based on the widely accepted “nearest-neighbor” determinant of the direction of apparent motions (Dawson, 1991), if the inducing ring point stop positions are displaced less than halfway across the gaps separating the test ring points, the expected apparent motion jump would be backward (counterclockwise) toward the preceding test ring points. If the inducing ring points stop exactly at the midpoint of the gaps between the test ring points the expected direction of any apparent motion jumps would be indeterminate. If the inducing ring points stop more than halfway across the gaps between the test ring points, the predicted apparent motion jumps would be forward (clockwise) toward the following test ring point, congruent with the actual inducing ring spin. The pattern in illusory motions that we observed—a gradual transition from a matching direction percept to a maximal reverse direction percept at the halfway point and then gradual return to a congruent percept—stands in stark contrast to this predicted pattern. The same inconsistency between the predicted pattern of motion cuing and observed pattern of motion biases will be present with a counterclockwise inducer rotation, and in displays in which the test ring precedes the inducing ring (with the roles of the test and inducing rings exchanged). The observed results therefore provide no indication that they are a consequence of apparent motion steps.

### The MAE

While in previous investigations of the MBE the illusory rotation of the test ring points have been primarily congruent with the inducing ring direction, the incidence of incongruent rotations has tended to increase in the course of a run of trials (Mattler & Fendrich, 2010). This increase in the frequency of incongruent motion reports is suggestive of an adaptation process, which brings to mind the motion direction reversals that occur with the classic MAE.

The MAE is an illusory motion in a stationary stimulus that is opposite the direction of a previously viewed adapting stimulus. It is generally thought to occur because the adapting stimulus fatigues neural units that mediate the perception of motion in a specific direction, giving an advantage to neural units that mediate motion in the opposing direction stimulus (see Anstis et al., 1998, for a review). This imbalance could arise at a processing stage as early as the retina (Barlow & Hill, 1963), although higher processing stages in V1 and extrastriate areas have also been implicated (Niedeggen & Wist, 1998).

While the buildup of incongruent illusory motion percepts in the course of MBE experimental runs is suggestive of an adaptation effect, we deem it very unlikely that the classic MAE contributes to the MBE direction reversals. If neural fatigue does play a role in the increasing incidence of reverse motion percepts, the nature of this fatigue would have to be fundamentally different from that which is thought to be the source of the MAE. This is the case because in all investigations of the MBE, trials with clockwise and counterclockwise rotations have been evenly intermixed. Any buildup of neural fatigue would therefore have to be selectively impairing neurons that mediate the biasing effect rather than motion percepts in particular spatial directions. The adapting periods that produce the MAE and MBE are also very different: while periods in the tens of *seconds* are typically needed to produce a MAE (e.g., Hershenson, 1993), the inducer durations used to produce the MBE can be as short as 30 ms (Mattler & Fendrich, 2010). In the experiments reported here, a 91 ms inducer duration was used—far shorter than one would expect to be needed to generate a conventional MAE.^1^ Finally, the temporal frequencies of the stimuli presented in MBE experiments exceed the temporal frequencies of the adapting stimuli that are effective in generating the MAE. Pantle (1974) reports that the duration of the MAE is highest when the adapter frequency is 1 to 5 Hz and starts to decline rapidly at 20 Hz, with no MAE occurring at 50 Hz. In the 2,250°/s conditions of the experiments presented here, reversed motion percepts were reported although the point positions along the inducing ring circumference were updated at 100 Hz.

### Biphasic Neural Responses

Several reported accounts of reversals in the normal direction of apparent motion percepts have argued that such reversals can be explained by biphasic neural responses occurring as early as the retina. Reversals in the direction of apparent motions were initially reported by Anstis (1970) who found that when a pattern is replaced by a slightly displaced photographic negative of itself the resulting apparent motion jump goes from the second stimulus to the first—opposite to its expected direction. Anstis and Rogers (1975) argue these reversals can be explained by the luminance profiles of the overlapping retinal receptive fields that process the contours of the successive patterns. Shiori and Cavanagh (1990) extended this account to explain motion direction reversals observed when a random pattern of black dots was shown displaced by a short distance if a short blank gray ISI was inserted between the presentations. They argue that the blank interval allows a “biphasic impulse response,” possibly originating in the retinal ganglion cells, to generate a reverse luminance representation of the preceding black dot pattern. This internally generated negative representation is taken to act like the inverted luminance images presented by Anstis and Rogers and therefore produce an apparent motion reversal akin to the one they found.

Several subsequent investigators have used this biphasic response hypothesis to account for other cases of apparent motion reversals (Mather, 2006; Pantle & Turano, 1992; Stout et al., 1994). Notably, in all of these cases the reverse motion percept requires a blank interval to be inserted between the frames of the apparently moving stimulus. Moreover, no reverse motion percept is seen if this blank ISI is dark. An earlier report by Braddick (1980) of reversals in the perceived direction of the stepped rotation of an annulus with bright and dark sectors preceded the formulation of this account but seems to fit with it since these reversals also required the insertion of a bright ISI between the annulus frames.

It does not appear, however, that a biphasic response explanation is applicable to the rotation reversals found with the MBE. This is the case because of the requirement that a luminant blank interval be shown for reversals of this kind to occur. Although a blank ISI between the inducing and test ring presentations is usually presented in MBE displays, both congruent and incongruent illusory rotations continue to be present when this ISI is completely eliminated (Mattler & Fendrich, 2010). Moreover, the ISI in MBE displays does not meet the core requirement that the blank be luminant: in *every* reported investigation of the MBE a *dark* ISI has been employed.

### The Interpretation of Competing Motion Signals by Perceptual Heuristics

Although the MBE's motion direction reversals cannot be attributed to the selective fatigue of neurons tuned to clockwise and counterclockwise rotations (see our discussion of the MAE) the more general premise that a competition between neural assemblies that mediate the perception of opposing motion directions can provide a useful framework for addressing the interplay of the MBE's congruent and incongruent motion percepts. We now speculate how such a competition might operate. Below we present an explanatory scheme that incorporates both the proposal by Stein et al. (2019) that the MBE involves a transformational apparent motion that may be related to the line motion illusion and the proposal by Mattler et al. (2021) that the MBE percept of a global rotation is facilitated by high-level cognitive processes.

The apparent motions produced by shifts in periodic gratings are inherently ambiguous since the retinal image change produced by a short grating shift in one direction can also be attributed to a longer shift in the opposite direction (see Pantle & Turano, 1992, for a discussion of this ambiguity). Morgan and Cleary (1992) address this directional uncertainty in a brief report on the ability of observers to correctly ascertain the direction of grating displacements. To account for the finding that when presented with identical stimuli the reports of different subjects ranged from errorless, to chance, to consistently in the wrong direction, Morgan and Cleary propose that grating displacements activated cortical Reichardt detectors tuned to both the short and long motion paths, and observers could attend to one or the other of the competing motion signals. We propose that in MBE displays when the test ring replaces the inducing ring there is a similar simultaneous activation of cortical motion detectors tuned to different motions. This supposition is based on the hypothesis, previously advanced by Stein et al. (2019), that when the ring transition occurs the segments of the inducing ring outline that flank each of the test ring points undergo a transformational apparent motion in which they contract into those points. These contractions can be regarded as bilateral instances of the shrinking line percepts reported by Downing and Treisman (1997) in their study of the line motion illusion. They give rise to opposing simultaneous rotations because the inducing ring segment on the counterclockwise side of each test ring point will contract clockwise while the segment on the clockwise side of each point will contract counterclockwise. Two questions that must then be addressed are why observers see a continuous rotation rather than these contractions and how the direction of this rotation is selected.

In their 2021 paper on the RRI, Mattler et al. propose the illusory rotation seen in both the RRI and MBE is produced by a heuristic that interprets the inducing to test ring transition as a consequence of the slowing down of a rapid rotation. This heuristic would account for the perception of a uniform rotation rather than a group of local contractions. The apparent motion linking process reported by Ramachandran and Anstis (1983) could provide low level support for this heuristic by yoking the direction the multiple contractions that occur along the inducing ring border. To explain the congruency effect and its reversal due to point misalignments we now posit this heuristic incorporates multiple stages: an initial monitoring stage that detects and extracts potentially relevant neural events, an interpretive stage which evaluates the current sensory input in the context of these events, and an implementation stage that determines the character of what is perceived given of this assessment.

In the case of the RRI, where the inducing ring is a truly continuous static outline circle, one would expect that the motion signals generated by a contraction of the contour segments on opposite sides of the test ring points to be balanced in strength. The motion signals extracted by the monitoring stage would therefore not favor a particular rotation direction, so it would be reasonable to predict a similar incidence of clockwise and counterclockwise rotation percepts. Contrary to this prediction, in their 2021 investigation, Mattler et al. found that with RRI displays a predominantly clockwise illusory rotation was seen. This suggests that in addition to low level stimulus-dependent signals the interpretive stage takes into account high level factors like an observer's familiarity with clockwise rotations.

**Figure 8.**
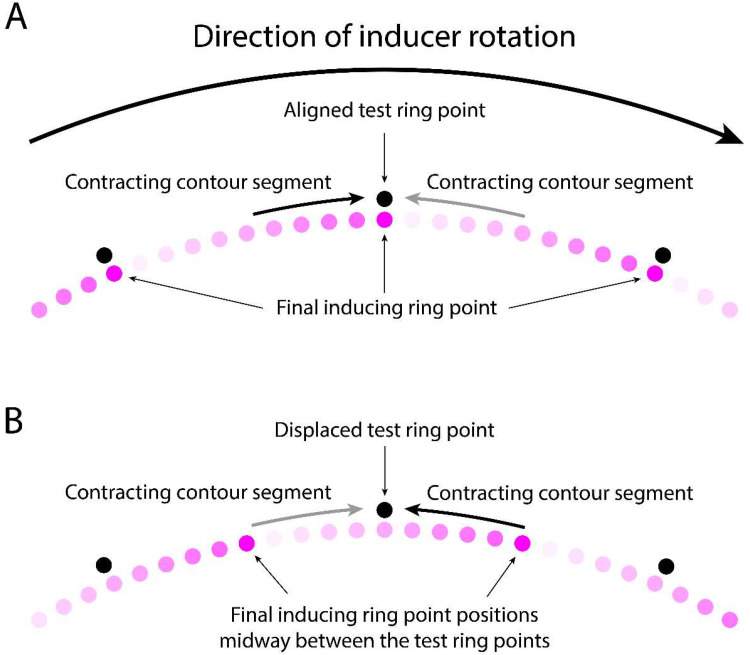
Direction and strength of the hypothetical contracting motions produced when the test ring replaces the inducing ring if the final inducing ring point locations are (A) aligned with the test ring points, and (B) midway between the test ring points. A clockwise inducing ring rotation is presumed. The shaded dots represent locations in the trail of persisting excitation behind the advancing points, with darker shades signifying greater temporal proximity to the appearance of the test ring points and consequently stronger motion signals. Note that the test ring points are only shown as vertically offset from the inducing ring points to allow the figure to depict them. A full explanation of this figure is presented in the discussion text.

In the case of the MBE, where the inducing ring outline is formed by the trail of retinal persistence produced by the advancing inducing ring points, two different information sources could give rise to directionally biased low level motion signals. One of these sources is the rotation of the inducing ring which, as Mattler and Fendrich first hypothesized in 2010, might be neurally encoded despite being inaccessible to consciousness. This biasing source could account for the direction congruency effect, but not the reversal of this effect by misalignments of the inducing and test ring points positions. The other potential source of a biasing signal would be an asymmetry in the strength of the opposing motion signals generated by the contraction of the inducing ring segments. An asymmetry of this kind might arise because the newly refreshed contour segments just behind the ring's advancing points will be temporally closer to the appearance of the test ring points than the contour segments just ahead of those points, which were refreshed earlier by the passage of the preceding point. In addition, the persisting retinal activity produced by the passage of an inducing ring point will start to fade as soon as the point has advanced past that location, so the persisting contour segments just behind the ring's advancing points are generated by a stronger retinal output than the contour segments in front of those points. Although in MBE displays the temporal difference in the time the contour segments behind and in front of the inducing ring are activated will normally be too small to produce a visually apparent contour intensity difference (with our 2,250°/s rotation rate it was only 10 ms), this temporal difference might nevertheless modulate the strength of the motion signals produced by the contour segment contractions, with the contraction of more recent segments generating stronger motion signals than the contraction of older segments. We will refer to this proposition as the *contraction-strength* hypothesis. It follows from this hypothesis that the contraction of the newly refreshed contour segment just behind each of the inducing ring points will produce a stronger motion signal than the contraction of the comparatively older faded segment in front of those points. Figure 8A illustrates these putative motion strength differences when the inducing and test ring points are aligned, and Figure 8B when these points are maximally misaligned.

As depicted in Figure 8A, when the inducing and test ring points are aligned the stronger contraction-based apparent motion signal will occur on the counterclockwise side of the test ring points and therefore have clockwise direction, matching the direction of the inducing rings rotation. This will be the case for every test ring point, so the joint effect of these local motion signals will produce a global motion signal that is congruent with the inducing ring's direction. In contrast, as depicted in Figure 8B, a misalignment of the inducing and test ring points will displace the more recently refreshed portion of the inducing ring contour to the clockwise side of the test ring points, so the stronger apparent motion signals will be counterclockwise, opposite to the direction of the inducing ring rotation. If the inducing ring rotation had been counterclockwise the same pattern of motion strength weightings would occur with the clockwise and counterclockwise motion directions reversed. Thus, the contraction-strength hypothesis is able to account for both, the motion congruency effect when the final inducing ring points are aligned with the test ring points and the reversal of the congruency effect when those point positions are maximally misaligned. Partial misalignments will presumably generate intermediate weightings of the congruent and non-congruent direction signals. How well these weighting changes map onto the observed motion congruency transitions remains to be determined.

Turning to displays in which the test ring precedes the inducing ring, a point-expansion counterpart to the contraction-strength hypothesis can be formulated to explain the perceived inducing ring start-up motions. However, when the inducing ring is presented before the test ring its disappearance allows the final state of its outline to be stored and assessed. When the inducing ring follows the test ring, all that will be stored when the test ring disappears is a persisting image of that ring's point positions, which will convey no inherently useful motion information.^2^ The perceived start-up motions must therefore be based on information registered during the brief period (10 ms when the inducing ring rotated at 2,250°/s, 15 ms when it rotated at 1,500°/s) during which the inducing ring outline is first being drawn.

**Figure 9.**
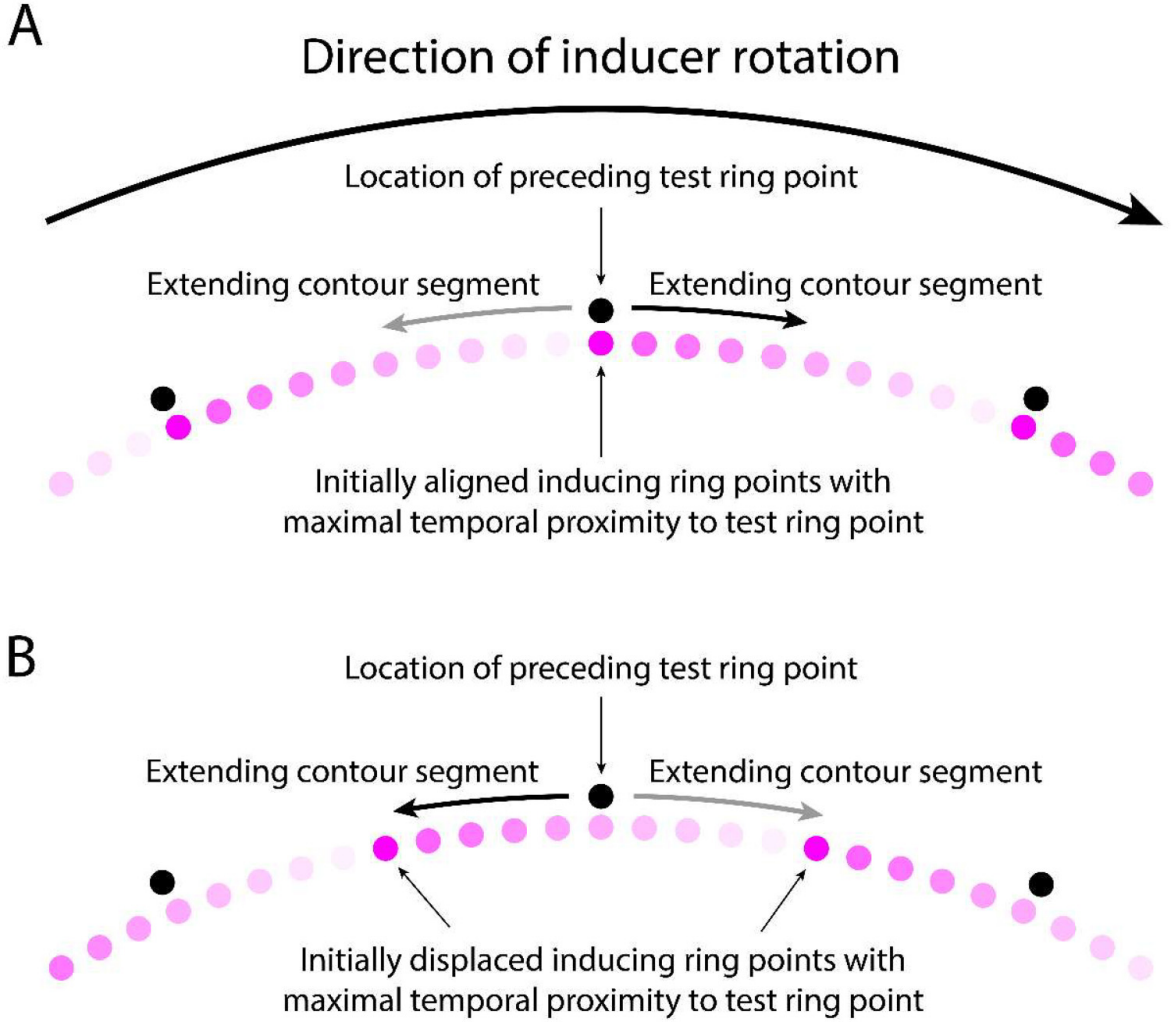
Direction and strength of the hypothetical point expansions when the test ring precedes the inducing ring if the initial inducing ring point locations are (A) aligned with the preceding test ring points, and (B) midway between the test ring points. A clockwise inducing ring rotation is presumed. The black dots show the positions where the test ring points had previously been displayed. Note they are only shown as vertically offset from the inducing ring points to allow the figure to depict them. The shaded dots represent locations in the trail of excitation behind the advancing points, with darker shades signifying a greater temporal proximity to the appearance of the preceding test ring points and consequently stronger motion signals. A full explanation of this figure is presented in the discussion text.

To account for these start-up motions and their alignment dependent reversal we posit that the persisting test rings point images are visually interpreted as expanding to form the inducing ring outline, with this expansion preferentially directed toward the inducing ring segments that are drawn first and therefore have the greatest temporal proximity to the test ring points. Figure 9 illustrates the expansion pattern that is expected given this premise, with Figure 9A showing the expected pattern when the test and inducing ring points are aligned, and Figure 9B showing the pattern when the inducing ring points appear midway between the test ring points, maximizing the misalignment of those points.

As shown in Figure 9A, when the inducing and test ring points are aligned, the initial contour segments drawn by the inducing ring points will extend forward from the persisting images of the test ring points on the side of those points that matches the rotation direction of the inducing ring (i.e., from their clockwise side when the rotation is clockwise). The proposed expansion of the points to form the inducing ring outline will therefore be fully congruent with the actual rotation and in accord with any motion signals generated by that rotation. The finding that the perceived start-up motion matches the actual rotation direction is therefore what one would expect.

In the misaligned case the situation is less straightforward. Figure 9B depicts the situation when the inducing ring points first appear displaced to locations midway between the test ring points. In this case the inducing ring contour segments drawn by those points will initially advance in the rotation direction toward the side of test ring points opposite the rotation direction (i.e., on their counterclockwise side when the rotation is clockwise). Consequently, the direction of the proposed expansion of the persisting test ring point images toward the growing contour segments will be opposite both the direction of the actual growth of these segments and the inducing ring's rotation direction. The finding that the reported start-up motion in this display condition is usually in this reversed direction suggests that the side of the test ring point images toward which the inducing ring points initially advance provides a motion cue that overrides the actual direction of the contour line expansion and any motion signals generated by the inducing ring rotation. While this outcome may seem counterintuitive, it accords with the fact that if no test points are presented the inducing ring rotation does not generate any start-up motion percept. In control conditions in which only the inducing ring is displayed what observers consciously experience is a static image of the inducing ring outline suddenly appearing in its entirety (e.g., Mattler & Fendrich, 2010). Previous investigations of the MBE have suggested that the rapid rotations of the inducing ring may be neurally registered despite the fact that they are not consciously visible (Mattler & Fendrich, 2010, Mattler et al., 2021), but given their invisibility it is not surprising that such motion signals have little or no ability to compete with other motion direction cues. Finally, we note that the stimulus sequence in test ring first MBE displays—the presentation of points followed by the presentation of lines—is the same as the sequence typically employed in line motion illusion displays. To the extent that both the proposed line contractions in MBE displays in which the inducing ring precedes the test ring and the proposed point expansions in MBE displays in which the test ring precedes the inducing ring involve transformational apparent motions linked to the line motion illusion, the neural basis of these two MBE variants will be similar despite the differences in our assumptions about the stimulus conditions that produce them.

### Summary and Conclusions

The MBE demonstrates the encoding of information about the rotation direction of a ring of points that spins so rapidly its rotation is not consciously detectable (Mattler & Fendrich, 2010). When this spinning “inducing ring” is followed by a “test ring” of stationary points, participants report an illusory ring rotation of the test ring. When the inducing ring is preceded by the test ring, the test ring is seen as launching into motion. In experiment 1 of this study, we demonstrate that the direction of the perceived rotations is dependent on whether the first or final inducing ring point positions are aligned with the test ring point positions. If these positions are the same, the perceived rotation direction tends to be congruent with the spinning ring's actual rotation. Progressively misaligning these positions produces a progressive tendency for the direction of the perceived test ring rotation to be opposite the inducing ring's direction, with incidence of these incongruent rotations reaching a maximum when the final inducing ring point positions fall midway between the test ring points. In experiment 2 we find a similar pattern, with the inducing ring appearing to launch into motion in its actual spin direction when the initial inducing ring point positions are aligned with the test ring point positions and launching into motion in the opposite direction of its actual rotation when those point positions are misaligned.

The present findings extend previous explanations of the MBE by showing the direction of the MBE illusory rotation is at least in part determined by the relationship between the inducing and test ring points at the moment the transition between these rings occurs. Explanations of this dependence based on apparent motion jumps between the point positions of the inducing and test rings, the MAE, and biphasic motion responses are ruled out. When the test ring follows the inducing ring, we propose a speculative alternative explanation in which the direction of the illusory rotation is determined by the balance between opposing signals generated when segments of the inducing ring that flank the test ring points contract to those points. When the inducing ring follows the test ring, we propose a speculative account in which persisting images of the test ring points expand to form the inducing ring outline with the direction of that expansion determined by the side of the test ring points where the inducing ring outline is initially drawn.

When they first described the MBE, Mattler and Fendrich (2010) argued that it demonstrated the encoding of motion information that the human visual system had not previously been considered capable of encoding. Subsequent investigations (Mattler et al., 2021; Stein et al., 2019) led to a modified account of the illusion which argues it involves two distinct processes: an apparent motion that produces the illusory test ring rotation and a separate direction biasing process that evaluates the available motion direction cues. The findings reported in this paper indicate that an updated view of the direction biasing process is needed since the direction of the illusory rotation is determined, at least in part, by an interaction between the inducing and test ring points when the transition between these rings occurs. Whether information about the inducing ring spin direction registered and stored *prior *to the ring transition also plays a role in determining the direction of the test ring rotation is a question that remains to be resolved. To address this question, we are currently conducting research in which the direction of the inducing ring rotation is being manipulated at different times in the course of the inducing ring presentation. To conclude, we note that while interpretations of the MBE have evolved and are continuing to evolve, it continues to be the case that the MBE is revealing aspects of human motion processing that have not been previously evident.

## Notes

1. Kanai and Verstraten (2005) have reported a rapidly developing form of the MAE they designate the rMAE. The rMAE was only observable, however, when dynamic test displays with ambiguous motions were employed. Moreover, although the rMAE developed far more quickly than conventional MAEs, when they employed adapting-stimulus to test-stimulus ISIs similar to those used in our MBE experiments it was only found when adapting periods were longer than 160 ms.

2. If the inducing and test ring points are initially aligned when the inducing ring points appear, the points of the two rings will overlap, so the test ring points will simply become the inducing ring points (momentarily blinking off when doing so if there is a long enough ISI). If the inducing ring points appear at positions that are not aligned with the test ring points apparent motion signals may be generated, but their expected directions will not be predictive of the rotation percepts actually observed (see the earlier section on “Direction Biasing by Apparent Motion Steps”).

## References

[bibr1-20416695251318945] Anstis S. M. (1970). Phi movement as a subtraction process. Vision Research, 10, 1411–1430. 10.1016/0042-6989(70)90092-1 5516541

[bibr2-20416695251318945] Anstis S. M. Rogers B. J. (1975). Illusory reversal of visual depth and movement during changes of contrast. Vision Research, 15, 957–961. 10.1016/0042-6989(75)90236-9 1166630

[bibr3-20416695251318945] Anstis S. Verstraten F. A. J. Mather G. (1998). The motion aftereffect. Trends in Cognitive Sciences, 2, 111–117. 10.1016/S1364-6613(98)01142-5 21227087

[bibr4-20416695251318945] Barlow H. B. Hill R. M. (1963). Evidence for a physiological explanation of the waterfall phenomenon and figural after-effects. Nature, 200, 1345–1347. 10.1038/2001345a0 14098503

[bibr5-20416695251318945] Braddick O. J. (1980). Low-level and high-level processes in apparent motion. Philosophical Transactions of the Royal Society of London. Series B Biological Sciences, 290, 137–151. 10.1098/rstb.1980.0087 6106234

[bibr6-20416695251318945] Burr D. C. Ross J. (1982). Contrast sensitivity at high velocities. Vision Research, 22, 479–484. 10.1016/0042-6989(82)90196-1 7112947

[bibr7-20416695251318945] Dawson M. R. (1991). The how and why of what went where in apparent motion: Modeling solutions to the motion correspondence problem. Psychological Review, 98, 569–603. 10.1037/0033-295X.98.4.569 1961774

[bibr8-20416695251318945] Downing P. E. Treisman A. M. (1997). The line-motion illusion: Attention or impletion? Journal of Experimental Psychology: Human Perception and Performance, 23, 768–779. 10.1037/0096-1523.23.3.768 9180044

[bibr9-20416695251318945] Hautus M. J. (1995). Corrections for extreme proportions and their biasing effects on estimated values of *d*′. Behavior Research Methods, Instruments, & Computers, 27, 46–51. 10.3758/BF03203619

[bibr10-20416695251318945] Hershenson M. (1993). Linear and rotation motion aftereffects as a function of inspection duration. Vision Research, 33, 1913–1919. 10.1016/0042-6989(93)90018-R 8249310

[bibr11-20416695251318945] Hsieh P.-J. Caplovitz G. P. Tse T. U. (2005). Illusory rebound motion and the motion continuity heuristic. Vision Research, 45, 2972–2985. 10.1016/j.visres.2005.02.025 15876447

[bibr12-20416695251318945] Kanai R. Verstraten F. A. J. (2005). Perceptual manifestations of fast neural plasticity: Motion priming, rapid motion aftereffect and perceptual sensitization. Vision Research, 45, 3109–3116. 10.1016/j.visres.2005.05.014 16023173

[bibr13-20416695251318945] Kelly D. H. (1972). Flicker. In Jameson D. Hurvich L. M. (Eds.), Visual psychophysics. Handbook of sensory physiology (Vol. 7/4, pp. 273–302). Springer. 10.1007/978-3-642-88658-4_11

[bibr14-20416695251318945] Macmillan N. A. Creelman C. D. (2005). Detection theory: A user’s guide (2nd ed.). Lawrence Erlbaum Associates.

[bibr15-20416695251318945] Mather G. (2006). Two-stroke: A new illusion of visual motion based on the time course of neural responses in the human visual system. Vision Research, 46, 2015–2018. 10.1016/j.visres.2005.12.022 16487987

[bibr16-20416695251318945] Mattler U. Fendrich R. (2007). Priming by motion too rapid to be consciously seen. Perception & Psychophysics, 69, 1389–1398. 10.3758/BF03192954 18078229

[bibr17-20416695251318945] Mattler U. Fendrich R. (2010). Consciousness mediated by neural transition states: How invisibly rapid motions can become visible. Consciousness and Cognition, 19, 172–185. 10.1016/j.concog.2009.12.015 20093045

[bibr18-20416695251318945] Mattler U. Stein M. Fendrich R. (2021). The ring rotation illusion: Properties and links of a novel illusion of motion. i-Perception, 12, 1–25. 10.1177/20416695211020019 PMC8191087 34164106

[bibr19-20416695251318945] Morey R. D. Rouder J. N. (2024). *BayesFactor*: *Computation of Bayes factors for common designs* [R package, version 0.9.12-4.7]. Retrieved from http://bayesfactorpcl.r-forge.r-project.org/

[bibr20-20416695251318945] Morgan M. J. Cleary R. (1992). Ambiguous motion in a two-frame sequence. Vision Research, 32, 2195–2198. 10.1016/0042-6989(92)90081-S 1304097

[bibr21-20416695251318945] Niedeggen M. Wist E. R. (1998). The physiologic substrate of motion aftereffects. In Mather G. Verstraten F. Anstis S. (Eds.), The motion aftereffect: A modern perspective (pp. 125–155). MIT Press.

[bibr22-20416695251318945] Pantle A. (1974). Motion aftereffect magnitude as a measure of the spatio-temporal response properties of direction-sensitive analyzers. Vision Research, 14, 1229–1236. 10.1016/0042-6989(74)90221-1 4428631

[bibr23-20416695251318945] Pantle A. Turano K. (1992). Visual resolution of motion ambiguity with periodic luminance- and contrast-domain stimuli. Vision Research, 32, 2093–2106. 10.1016/0042-6989(92)90071-P 1304087

[bibr24-20416695251318945] Ramachandran V. S. Anstis S. M. (1983). Perceptual organization in moving patterns. Nature, 304, 529–531. 10.1038/304529a0 6877373

[bibr25-20416695251318945] Shiori S. Cavanagh P. (1990). ISI produces reverse apparent motion. Vision Research, 30, 757–768. 10.1016/0042-6989(90)90101-P 2378068

[bibr26-20416695251318945] Stein M. (2020). *Spatial and temporal dependencies of the motion bridging effect: Investigations of an illusory motion* [Doctoral dissertation]. Georg-August Universität School of Science. http://dx. 10.53846/goediss-7977

[bibr27-20416695251318945] Stein M. Fendrich R. Mattler U. (2019). Stimulus dependencies of an illusory motion: Investigations of the motion bridging effect. Journal of Vision, 19, 1–23. 10.1167/19.5.13 31100129

[bibr28-20416695251318945] Stein M. Fendrich R. Mattler U. (2020). Encoding information from rotations too rapid to be consciously perceived as rotating: A replication of the motion bridging effect on a liquid crystal display. i-Perception, 11, 1–11. 10.1177/2041669520925111 PMC7249587 32547723

[bibr29-20416695251318945] Stout J. J. Pantle A. Mills S. L. (1994). An energy model of interframe interval effects in single-step apparent motion apparent motion. Vision Research, 34, 3223–3240. 10.1016/0042-6989(94)90086-8 7975353

[bibr30-20416695251318945] Tse P. U. Cavanagh P. Nakayama K. (1998). The role of parsing in high-level motion processing. In Watanabe T. (Ed.), High-level motion processing: Computational, neurobiological, and psychophysical perspectives (pp. 249–266). MIT Press.

